# Myocardial Infarction in a 28-Year-Old Male With Neurofibromatosis Type 1

**DOI:** 10.7759/cureus.11254

**Published:** 2020-10-30

**Authors:** Mitra Patel, Dipen Patel, Christian Nehme, Amala Ambati, Carson E Oostra

**Affiliations:** 1 Internal Medicine, University of Toledo College of Medicine, Toledo, USA; 2 Internal Medicine, University of Toledo Medical Center, Toledo, USA

**Keywords:** neurofibromatosis, stemi, coronary aneurysm

## Abstract

Neurofibromatosis type 1 (NF1) is an autosomal dominant genetic disorder that affects multiple systems throughout the body. Although there are multiple documented vasculopathies that can be seen in NF1, there are very few documented cases of coronary artery aneurysms with complete thrombosis of the ectatic vessel resulting in myocardial infarction. This case report describes a 28-year-old male with a past medical history of NF1 who presented with an anterolateral ST-segment elevation myocardial infarction. He underwent urgent cardiac catheterization, which was significant for severe thrombotic occlusion of the mid-left anterior descending artery (LAD) with thrombolysis in myocardial infarction (TIMI) flow 0. The LAD was noted to be severely ectatic. Percutaneous coronary intervention (PCI) with thrombectomy was attempted and was unsuccessful, with TIMI flow 0 after the intervention attempt. An echocardiogram was performed, which showed left ventricular ejection fraction (LVEF) of 30%-35%. This case report is presented to familiarize physicians with the rare vasculopathies that can occur in patients with NF1. Occlusive or aneurysmal disease can occur almost anywhere in the body in patients with NF1 due to the proliferation of fusiform endothelial cells in the blood vessels.

## Introduction

Neurofibromatosis type 1 (NF1) is typically an inherited autosomal dominant disorder in the NF1 tumor suppressor gene, which can predispose patients to benign and malignant tumors of the nervous system. However, up to 50% of patients with NF1 do not have any family history and acquire the disease from spontaneous mutations. Clinical features of the disease include café-au-lait macules, axillary or inguinal freckling, neurofibromas, lisch nodules, osseous lesions, or an optic pathway glioma [1]. Patients may develop aneurysmal arterial diseases or occlusive arterial diseases in the heart or peripheral vasculature. The literature on coronary artery vasculopathies in NF1 is scant. Vasculopathy in NF1 patients has involved cerebral, renal, and aortic vessels [2]. Published cases of myocardial infarction in NF1 patients points to several different mechanisms ranging from occlusive disease, vasospasm, or coronary artery aneurysms [3,4]. Researchers speculate that the underlying pathological changes resulting in vasculopathies in NF1 patients appears to be angiodysplasia, such as intimal smooth muscle proliferation, neoangiogenesis, and plexiform/angiomatoid intimal proliferation [4].

## Case presentation

**Figure 1:**
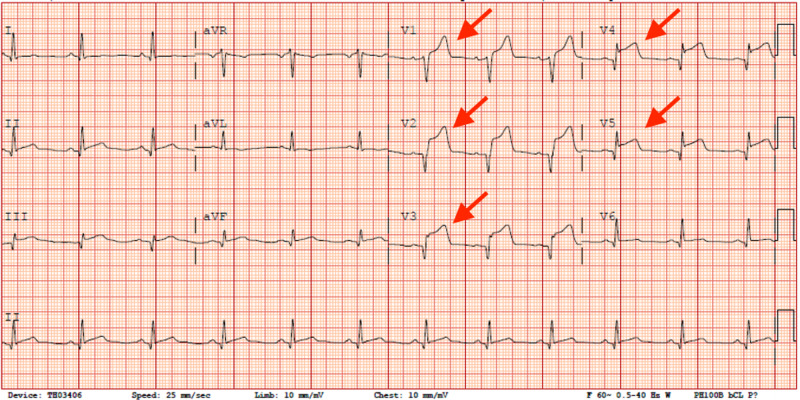
Initial EKG prior to coronary intervention expressing ST-segment elevations in leads V1-V5

A 28-year-old male presented with dull, left-sided, substernal chest pain. On physical exam, he was found to be alert, oriented, and cooperative. His lungs were clear to auscultation and there was no use of accessory muscles of respiration. On cardiac exam, his heart rate and rhythm were regular without any murmurs, rubs, or gallops and a normal S1 and S2 heart sounds. His initial EKG showed 4-5mm ST-segment elevations in leads V1-V5 (Figure 1). The initial troponin level was 35.50. He has a past medical history of NF1.

**Figure 2:**
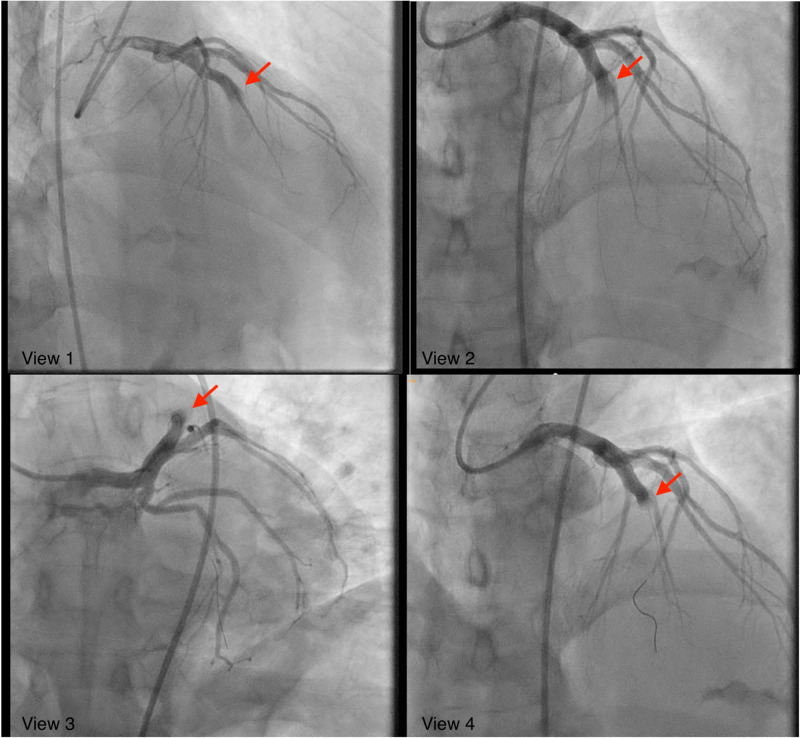
Ectatic dilation of the left anterior descending coronary artery with obstruction and lack of distal blood flow as seen on cardiac angiography

The patient’s troponin continued to trend upwards to 49.27 and peaked at 62.01. CK-MB levels were not performed. He was initially given nitroglycerin, aspirin, and started on heparin. Cardiac catheterization was performed and was significant for severe thrombotic occlusion of the mid-left anterior descending artery (LAD) with TIMI flow 0. The time from the patient’s initial symptoms to balloon was approximately two hours. PCI w/ thrombectomy was attempted and was unsuccessful as the operator was unable to clear the thrombus, with TIMI flow 0 after the intervention attempt. The LAD was noted to be severely ectatic (Figure 2).

**Figure 3:**
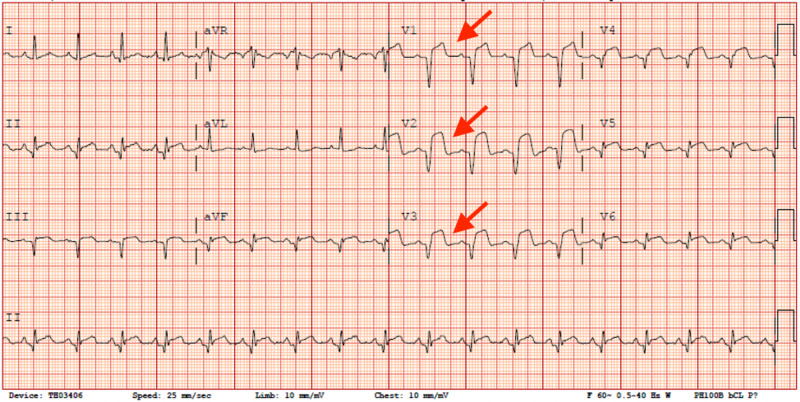
EKG after failed revascularization after coronary intervention

The unsuccessful percutaneous coronary intervention (PCI) was likely due to complete thrombotic occlusion of the vessel due to aneurysmal dilatation. The mid-inferior, apical-anterior, and apical-inferior segments of the LV were akinetic on angiography. Left ventricular end-diastolic pressure (LVEDP) was 23. After cardiac catheterization, the patient was started on tirofiban and dual antiplatelet therapy. An echocardiogram was performed, which showed a left ventricular ejection fraction (LVEF) of 30-35% and LV hypokinesis with no clot formation. He had multiple episodes of nonsustained ventricular tachycardia (NSVT) the day after his catheterization. Due to low LVEF, the patient was placed on a life vest. His medical therapy was then optimized with the addition of a beta-blocker, ACE inhibitor, and spironolactone to his regimen throughout his hospital course. EKG after coronary intervention showed a slight decrease in amplitude of ST-segment elevations (Figure 3).

LVEF on follow-up echocardiogram was 30%-35% and the patient received an implantable cardioverter defibrillator (ICD) device. After reviewing his echocardiogram, we believed that the image was missing a good portion of his apex. Ventriculogram during cardiac catheterization suggested aneurysmal behavior of the left ventricle. A cardiac MRI (CMR) was performed to assess this suspicion and showed thrombus formation in the left ventricular apex. A hematologist assessed the patient due to concerns of a clotting disorder and determined there was no underlying clotting disorder. There was no evidence of testosterone or recreational drug use, including cocaine and methamphetamines. There was no evidence of rheumatologic disease, autoimmune disease, infective arthritis, hyperviscosity, Buerger’s disease, or Raynaud's. There was no family history of early coronary artery disease or thromboembolism and no history of radiation or stem cell transplant. It was believed that his coronary artery aneurysm was most likely related to his existing NF1.

## Discussion

NF1 is frequently associated with neurological symptoms. However, numerous different organ systems can be affected by this disease making syndromal identification difficult. Patients can suffer from relatively benign neurofibromas to deforming skeletal abnormalities to malignant cancers such as breast cancer and leukemia.

Identification and reporting of cardiovascular symptoms in NF1 patients are rare. Studies using echocardiograms have shown “that up to 27% of NF1 patients have a cardiac anomaly" [1]. Congenital heart disorders have also been associated with NF1 [1]. Renal artery stenosis and subsequent hypertension is one of the most common vascular manifestations of NF1. Another vascular manifestation is concomitant coarctation of the abdominal aorta [3]. Lie described intimal arterial proliferation resulting in abdominal coarctation of the aorta and renal artery stenosis in NF1 patients [4]. Other NF1 patients can develop arteriovenous malformations [1].

In 1994, Lie reported “premature arteriosclerosis obliterans” in a 19-year-old male with NF1 who had to have a left below-knee amputation. Angiodysplasia was observed in virtually all of this patient’s arterial samples. While vasculopathies of NF1 may resemble systemic vasculitis, biopsy results show that angiodysplasia rather than inflammation of blood vessels is responsible for the vasculopathies in NF1 patients [4]. Some researchers speculate that defects in the NF1 gene result in increased proliferation and growth of vascular endothelial cells [1].

When vasculopathies develop in NF1 patients, they can have severe, or worse, deadly consequences. Kanter and colleagues report cases of NF1 children as young as 22 months and 6 years old who have suffered sudden cardiac death after being found in ventricular fibrillation [5]. Fuchi and colleagues describe a case of a 23-year-old male with NF1 who had a myocardial infarction due to “severe organic stenosis in the left anterior descending artery, and an ectatic left circumflex artery” [2]. Daley and Rubinstein describe a case of myocardial infarction and coronary artery aneurysm in an NF1 patient [3]. Babinska and colleagues describe a case of myocardial infarction in a patient with NF1 and pheochromocytoma [6].

Further research establishing the causal mechanism of vasculopathy in NF1 patients is needed. Future research studies should try to quantify the correlation between NF1 disease and cardiovascular vasculopathies. Moreover, a review article of myocardial infarctions in NF1 is warranted.

## Conclusions

NF1 is an autosomal dominant genetic disorder that affects multiple systems throughout the body. Although there are multiple documented vasculopathies that can be seen in NF1, there are very few documented cases of coronary artery aneurysms with thrombosis of the ectatic vessel resulting in myocardial infarction. The low incidence, lack of clinician awareness, and severity of a life-threatening event in a young individual can make diagnosis and management challenging.
